# Sex-Dependent Aggregation of Tinnitus in Swedish Families

**DOI:** 10.3390/jcm9123812

**Published:** 2020-11-25

**Authors:** Natalia Trpchevska, Jan Bulla, Matilda Prada Hellberg, Niklas K. Edvall, Andra Lazar, Golbarg Mehraei, Inger Uhlen, Winfried Schlee, Barbara Canlon, Silvano Gallus, Jose Antonio Lopez-Escamez, Christopher R. Cederroth

**Affiliations:** 1 Laboratory of Experimental Audiology, Department of Physiology and Pharmacology, Karolinska Institute, 171 77 Stockholm, Sweden; natalia.trpchevska@ki.se (N.T.); matilda.prada.hellberg@gmail.com (M.P.H.); niklas.edvall@ki.se (N.K.E.); barbara.canlon@ki.se (B.C.); 2 Department of Mathematics, University of Bergen, 5020 Bergen, Norway; Jan.Bulla@uib.no; 3 Department of Psychiatry and Psychotherapy, University of Regensburg, 93053 Regensburg, Germany; winfried.schlee@gmail.com; 4 Hörsel och balansmottagningen, Karolinska Universitetssjukhuset, 171 76 Stockholm, Sweden; andra.lazar@sll.se (A.L.); inger.uhlen@sll.se (I.U.); 5 Decibel Therapeutics Inc., Boston, MA 02215, USA; gmehraei@gmail.com; 6 Department of Environmental Health Sciences, Istituto di Ricerche Farmacologiche Mario Negri IRCCS, 20156 Milan, Italy; silvano.gallus@marionegri.it; 7 Otology & Neurotology Group, Department of Genomic Medicine, Pfizer—Universidad de Granada—Junta de Andalucía Centro de Genómica e Investigación Oncológica (GENYO), PTS, Avenida de la Ilustración 114, 18016 Granada, Spain; antonio.lopezescamez@genyo.es; 8 Department of Otolaryngology, Hospital Universitario Virgen de las Nieves, Instituto de Investigacion Biosanitaria ibs.GRANADA, 18012 Granada, Spain; 9 National Institute for Health Research (NIHR) Nottingham Biomedical Research Centre, Nottingham University Hospitals NHS Trust, Ropewalk House, Nottingham NG1 5DU, UK; 10 Hearing Sciences, Division of Clinical Neuroscience, School of Medicine, University of Nottingham, Nottingham NG7 2UH, UK

**Keywords:** tinnitus, familial aggregation, genetic risk, hearing loss, bilateral, unilateral, constant

## Abstract

Twin and adoption studies point towards a genetic contribution to tinnitus; however, how the genetic risk applies to different forms of tinnitus is poorly understood. Here, we perform a familial aggregation study and determine the relative recurrence risk for tinnitus in siblings (λs). Four different Swedish studies (N = 186,598) were used to estimate the prevalence of self-reported bilateral, unilateral, constant, and severe tinnitus in the general population and we defined whether these 4 different forms of tinnitus segregate in families from the Swedish Tinnitus Outreach Project (STOP, N = 2305). We implemented a percentile bootstrap approach to provide accurate estimates and confidence intervals for λs. We reveal a significant λs for all types of tinnitus, the highest found being 7.27 (95% CI (5.56–9.07)) for severe tinnitus, with a higher susceptibility in women (10.25; 95% CI (7.14–13.61)) than in men (5.03; 95% CI (3.22–7.01)), suggesting that severity may be the most genetically influenced trait in tinnitus in a sex-dependent manner. Our findings strongly support the notion that genetic factors impact on the development of tinnitus, more so for severe tinnitus. These findings highlight the importance of considering tinnitus severity and sex in the design of large genetic studies to optimize diagnostic approaches and ultimately improve therapeutic interventions.

## 1. Introduction

Subjective tinnitus is the phantom perception of sound in the absence of a corresponding external acoustic stimulus. As there are no objective measures, the prevalence of tinnitus has always been self-reported and varies between 5.1 to 42.7% depending on its definition [1]. However, a rarer form is severe tinnitus, which affects 1–3% of the population to the point of impacting quality of life [2,3]. Recent studies converge towards the notion that subjective tinnitus results from a maladaptive plasticity in response to sensory deprivation, such as hearing loss [4]. Damage in the hearing system (the cochlea) leads to central neural compensatory responses (central gain) along the auditory pathway, something which is observed in both animals and humans [5]. In addition, limbic structures (e.g., amygdala) have been found to interact with the auditory pathway in animals and humans with tinnitus and is thought to exacerbate tinnitus-related hyperactivity [6]. Recently, it has been suggested that the lack of effective treatment may be due to the heterogeneity of tinnitus [7], whose subtypes remain to be established [8].

Tinnitus is mainly considered a symptom and has been thought to derive only from environmental influences. A handful of studies have made attempts to reveal genetic influences, with mixed results [9]. For instance, a large multicenter study with 198 families showed that the risk of having tinnitus when having another member of the family with tinnitus was 1.7 [10]. Another population-based family study (*n* = 28,066 participants) determined a heritability of H_2_ = 11% [11]. We previously speculated that possible reasons for these failures may have resided in poor definitions of tinnitus [12]. In a twin study, when considering laterality as a potential classification of tinnitus, a heritability of 68% was found for bilateral tinnitus in men, while in contrast, unilateral tinnitus showed a heritability of 27%, suggesting a greater involvement of genetic factors in the former [13]. As twin studies may still show bias due to uncontrolled shared-environment effects [14], we undertook an adoption study based on national medical registry data in which tinnitus was clinically diagnosed by a medical doctor. The odds ratio (OR) for tinnitus was 2.01 (95% CI, 1.10–3.69) for adoptees of affected biological parents, whereas the OR for adoptees with an affected adoptive parent was 1.04 (95% CI, 0.53–2.04) adjusted for age, sex, county, educational attainment, depression, anxiety, and hearing loss, confirming that the familial transmission of clinically significant tinnitus is genetically influenced (H_2_ = 31%) and not due to shared-environment [15]. These two studies support a genetic contribution to tinnitus in families. Indeed, Clifford et al. recently published the first large genome-wide association study using the UK Biobank and replicated 3 loci and 8 genes in the Million Veterans Program with a single-nucleotide variants (SNP) heritability of 6.3% [16], providing a first basis of tinnitus as a neurological disorder [17].

Here, we used familial clustering as an analysis to estimate the relative sibling risk for tinnitus. Familial aggregation studies are used in genetic epidemiology to estimate recurrence risk of a trait within a family, which is the initial step to identify hereditary conditions [18]. The general approach is to determine whether having a relative with tinnitus increases one’s risk of developing tinnitus. This is assessed by a recurrence risk ratio or sibling recurrence risk (lambda sib [λs]). λs is defined as the risk to siblings of probands (the sample individual) for a specific condition, relative to the population prevalence (see Supplementary Materials 1) [19]. λs has been used to estimate the genetic risk for a wide range of diseases or traits. For instance, in schizophrenia, the sibling recurrence rate is 9% whereas the prevalence in the population is 1% (λs = 9).

## 2. Experimental Section

### 2.1. Participants of STOP

Participants were invited to join the Swedish Tinnitus Outreach Project (STOP) via social media channels and through partnerships with the Karolinska Hospital and local cohorts, including LifeGene [20]. All participants above 18 years of age were eligible. Voluntary registration was done on a website from STOP (https://stop.ki.se). After providing informed consent for having their data stored in a database and analyzed, participants were invited to fill an online questionnaire, which was answered between November 2015 and January 2018. The project has been approved by the local ethics committee “*Regionala etikprövningsnämnden*” in Stockholm (2015/2129-31/1).

We used the following question #A17 from the ESIT-SQ [21] for categorizing participants with tinnitus, who answered positively on the question that reads: “*Tinnitus refers to the perception of noise in your head or ears, such as ringing or buzzing, in the absence of any corresponding source of sound external to your head. Over the past year, have you had tinnitus in your head or in one or both ears that lasts for more than 5 min at a time*?” with response options “Yes, most or all of time; Yes, a lot of the time; Yes, some of the time; No, not in the past year; No, never; Do not know”. Out of 2457 participants who responded positively to the #A17 tinnitus question, 1185 were males and 1261 females. Three subjects reporting “Intersex” and 8 reporting “Prefer not to say” were excluded. In order to focus the study on subjective tinnitus, participants answering “yes” to the following ESIT-SQ question #B17 “Has a clinician ever heard your tinnitus?” were defined as objective tinnitus and thus were also excluded (141 participants). Our final study sample includes 2305 participants.

### 2.2. Tinnitus Subtyping

Subtyping was performed using the ESIT-SQ questionnaire to categorize subjective tinnitus based on laterality, severity, and whether it is constant or not. *Laterality of* tinnitus was defined based on the question #B15 asking “*Where do you perceive your tinnitus?*” with response options “Right ear”, “Left ear”, “Both ears, worse in right”, “Both ears, worse in left”, “Both ears, equally”, “Inside head”. Bilateral tinnitus was defined with the response options “Both ears, worse in right”, “Both ears, worse in left”, and “Both ears, equally”. Conversely, unilateral tinnitus was defined by the response options “Right ear”, and “Left ear”. Information on *constant* tinnitus was obtained based on participants answering “Constant” to the question #B2 “*What best describes your tinnitus during the day?*” with possible answers: “ Constant: you can always or usually hear it in a quiet room”, “Intermittent: comes and goes”, and “Cannot always hear it in quiet room”. Question #B4 addresses severity by asking “*Over the past year, how much does your tinnitus worry, annoy or upsets you when it is at its works*?” with response options “Severely”, “Moderately”, “Slightly”, and “Not at all”. Participants answering “Severely“ were considered in the severe tinnitus group.

### 2.3. Estimation of the Prevalence of Specific Tinnitus Subgroups in the Population

The prevalence in the adult (>18 years of age) general population of bilateral tinnitus was calculated using data from the Swedish Twin Registry (STR; N = 67,615) [22]. For unilateral tinnitus, the same dataset was used, but an age-correction procedure was adopted such that the sample would match the age range of the unilateral group from STOP (STR; N = 59,507). Details on the age-correction procedure are available in Supplementary Materials 1. The prevalence of constant tinnitus was estimated using LifeGene after an age-correction procedure (N = 26,696) [20]. Prevalence of severe tinnitus was found using data from both one wave (2010) of the Stockholm Public Health Cohort (SPHC, N = 72,295) [23] and one wave of SLOSH (year 2006, N = 19,992) [24].

### 2.4. Familial Aggregation

The estimation of the familial aggregation was performed using the #A8 question from ESIT-SQ that we subsequently modified to include information on the number of relatives: “How many first-degree relatives (parents, children, siblings) do you have and how many of them have do you know to have tinnitus or hearing loss?”, with potential answers for the three categories separately. Only siblings were investigated to estimate familial aggregation (Supplementary Materials 2).

The recurrence risk ratio for family members was used to estimate familial aggregation, or clustering. The recurrence risk is defined as the probability of a relative of an affected individual also to be affected [25]. When the recurrence risk within family members is compared to the prevalence in the general population, a recurrence risk ratio is obtained. The recurrence risk ratio is depicted as the lambda score (*λ*). It measures the combined risk ratio of all involved locus and is, therefore, a good measurement for genetic heterogeneity [19,26,27]. A *λ* greater than one indicates that the risk of an individual is increased if a relative is affected by the condition, i.e., that there is familial aggregation for this condition [28]. Commonly the *λ* for siblings and offspring is used for the estimation of the total recurrence risk ratio [29]. These are first degree relatives (FDR) and share a high degree of genetic material with the probands. Thus, should such clustering exist for the condition, it would be evidenced in FDR calculations.

The recurrence risk ratio, λ, is calculated as such:

$$\lambda =\frac{K_{r}}{K}$$

where $K_{r}$ is the prevalence of the condition within the group of family members, known as the recurrence risk, and K corresponds to the prevalence in the population. The quantity $K_{r}$ is calculated as follows:

$$K_{r}=\frac{no.\;of\;affected\;relatives\;of\;affected\;probands}{total\;no.\;of\;relatives\;of\;affected\;probands}$$

We estimated λ by assessing the risk among family members (i.e., siblings) with any tinnitus and subtypes of tinnitus, compared with the population prevalence. It should be noted that the population prevalence is not derived from a population in the statistical sense but is also an estimate subject to uncertainty.

### 2.5. Statistical Analysis

Calculation of the confidence intervals for the population prevalence (Table 1) as well as the lambda scores and corresponding confidence intervals (Table 2) was carried out with the statistical software package R version 3.6.0. The confidence intervals for the population prevalence result from Wilson’s score-test-based interval approximation for binomial proportions (https://www.tandfonline.com/doi/abs/10.1080/00031305.1998.10480550). We present details on the implemented procedures for the lambda score and related *p*-values in Supplementary Materials 1.

## 3. Results

We aimed to quantify the familial aggregation of tinnitus by estimating λs for different forms of tinnitus, namely, bilateral or unilateral, constant, and severe, using data from 2446 participants from the Swedish Tinnitus Outreach Project (STOP). The challenge was to identify estimates for these different forms of tinnitus in the general Swedish population. We identified four large national studies, the combination of which allowed us to determine the prevalence for bilateral, unilateral, constant, and severe tinnitus (Table 1). Sex prevalence estimates showed a clear bias towards greater prevalence in males for all tinnitus subtypes.

We next estimated the λs for all potential subtypes of tinnitus by comparing prevalence of tinnitus within participants from the Swedish Tinnitus Outreach Project who were recruited for the purpose of tinnitus subtyping studies (Table 2). We could not identify articles in which λs included confidence intervals and *p*-values taking uncertainty in both the population and the familial prevalence estimates into account. Hence, we implemented a percentile bootstrap approach to provide accurate estimates and significance values for the calculated λs (see Supplementary Materials 1). The recurrence ratios for participants with bilateral was λs_Bil_ = 1.79 (95% CI (1.55–2.04)) and did not differ from that of unilateral tinnitus λs_Unil_ = 1.99 (95% CI (1.45–2.56)). The λs of constant tinnitus increased to 2.29 (95% CI (2.01–2.58)). Interestingly, with increasing severity, tinnitus showed the highest lambda scores, reaching 7.27 (95% CI (5.56–9.07)) for severe tinnitus.

With a sex-specific bias in the prevalence of these different forms of tinnitus, we performed sex stratified analyses. In contrast to our expectations, we found consistently higher λs for women than for men, although being significant only for constant tinnitus (Constant T♂: 1.58 (1.31–1.86); Constant T♀: 3.32 (2.75–3.92)) and severe tinnitus (Severe T♂: 5.03 (3.22–7.01); Severe T♀: 10.25 (7.14–13.61)). The sexual dimorphism was also obvious for bilateral tinnitus, although not being significant between the two sexes (Bilateral T♂: 1.50 (1.23–1.78); Bilateral T♀: 2.16 (1.74–2.60)). Overall, the estimated recurrence risk ratios appear to be greater in women for some types of tinnitus tested here, suggesting a greater genetic susceptibility in women in particular to constant and severe and potentially bilateral tinnitus.

## 4. Discussion

The present study reveals that the greatest recurrence risk for siblings occurs in subjects with severe tinnitus. These findings are consistent with our recent adoption study using national registry-based data revealing a genetic contribution to the familial transmission of “clinically significant” tinnitus and a lack of shared-environment effects [15]. Since a large proportion of subjects have tinnitus severe enough to seek medical support, it is thus very likely that the generation of severe tinnitus is genetically influenced, more so than any other form of tinnitus. Although the adoption study could not reveal sex differences in tinnitus liability due to limitations in the sample size, the present work suggests that a greater risk occurs in women.

We previously revealed in a twin study a greater heritability for bilateral tinnitus in men (H_2_: 68% (95% CI: (63–73))) when compared to women (H_2_: 41% (95% CI: (23–58))) [13]. Noteworthy, this gender pattern was inverted (62% in females, 22% in males) when focusing on younger groups (<40 years of age), albeit not reaching significance likely due to sample size limitations [13]. Here, 27.4% of the participants were below 34 years of age, suggesting that young age may be an important contributor to phenotypic expression of genetically transmitted tinnitus in women. In this regard, while the prevalence of tinnitus is greater in men, there are increasing reports showing that tinnitus is more bothersome and psychologically impactful in women [1,30,31]. It is thus possible that the development of constant and/or severe tinnitus is more genetically influenced in women than in men. The sibling recurrence risk observed here in women with severe tinnitus (9.73) is in the range of what has been found for schizophrenia (9) and bipolar disorders (7.9) [32]. However, the sex bias we observe for tinnitus suggests a strong influence of sex in the pathophysiology of constant and severe tinnitus. The results of this study have substantial implications for further genetic studies mapping gene variants related to tinnitus, indicating that sex and severity are key elements of a subtype that is genetically determined. A recent large genome-wide association study only revealed a handful of loci associated with a broad definition of tinnitus [17], but the selection of severe cases and stratification by sex may lead to the identification of novel pathways with a sex-specific involvement in the pathophysiology of tinnitus. This approach has been proven effective in elucidating the genetic landscape of autism [33], bipolar disorder [34], major depressive disorder [35], and obsessive-compulsive disorder [36].

Emerging evidence suggests serotonin could play a role in the mechanism of tinnitus severity. The 5-HTTLPR polymorphism in the promoter of the serotonin transporter gene *SLC6A4* was found associated with greater tinnitus severity [37]. Although no studies have replicated these findings, a pilot GWAS on tinnitus showed an enrichment in serotonin signaling but without identifying genome wide significant variants due to limited sample size [38]. In support of the potential contribution of serotonin to tinnitus severity, serotonin has been shown to enhance signaling only from the multisensory input in the dorsal cochlear nucleus (DCN), while decreasing input from auditory fibers [39]. More specifically, serotonin increased excitability and spontaneous firing of vertical cells, leading to a vertical cell-mediated inhibitory activity in fusiform cells. Such mechanism could underlie the enhanced multisensory processing in the DCN observed during tinnitus in animal models [40,41], and whose bimodal somatosensory and auditory stimulation can decrease the severity of tinnitus [42]. Additional studies are needed to clarify the role of serotonin on tinnitus severity.

The present findings showing a high familial transmission of severe tinnitus may explain the low heritability of tinnitus reported in previous studies [10,11]. In one of the studies, tinnitus was defined as “*Nowadays, do you ever get noises in your head or ear (tinnitus) which usually last longer than five minutes?*”, which is very broad and may encompass a large number of subtypes and thus overestimate prevalence. The second one used the question “*Are you bothered by ringing in your ears?*”, which does not specifically define whether tinnitus is constantly and presently perceived, nor whether it is severe. Thus, the inclusion of separate items for the present percept, severity and duration is important to consider when classifying tinnitus.

To our knowledge, there are no studies providing recurrence risk ratio along with measures of dispersion (e.g., CI, *p*-value) taking uncertainty in both population and familial prevalence estimates into account. We provide here a bootstrap-based methodology allowing to generate such values when the ratio uses two distinct data sources for population and familial prevalence estimates—an approach that can be used in other diseases. Indeed, estimates of the recurrence risk ratio are highly dependent on available prevalence estimates in the population. Previous systematic reviews have revealed the prevalence for constant, or severe tinnitus, however the reported values varied a lot between studies likely due to the formulation of the questions addressing tinnitus, but also regional effects [1]. Thus, the use in the present study of different national cohorts with data on specific subtypes (laterality, severity, and whether it is constant or intermittent) is a major strength, something that cannot be achieved with the current medical registry data codes for tinnitus.

This study has several limitations. The first one is the ecological design of the study: Data are analyzed at the population or group level, rather than at the individual level. Prevalence of different types of tinnitus in the general population is computed from a set of large studies; the same is computed among siblings of tinnitus subjects; finally, the two prevalence estimates are compared. The source for these estimates is different with potentially differences in mean age and differences in the assessment of tinnitus. Furthermore, due to the different sources of population and familial estimates, it was not possible to control for chronic ear diseases that could have contributed to tinnitus. In this regard, information on hearing (e.g., audiometry, or self-reported hearing ability) is also missing, but given the study design, it would require similar individual audiometric or hearing information within each population study and the one in which sibling risk is estimated. In tinnitus studies, it is important to consider auditory measures beyond the clinical standard which is set at 8 kHz [43]. Many individuals have normal hearing thresholds up to 8 kHz, but 84.8% of these (*n* = 589) show elevated thresholds (>20 dBHL) at higher frequencies when assessed up to 16 kHz (C.R.C., unpublished data). The second is a bias in recall whereby 27.2% of participants could not recall whether they have a family member that is affected. In the cases where affected sibling(s) were reported, there is no information whether the tinnitus type matches the one of the proband, nor whether the affected siblings were clinically diagnosed, or their tinnitus was self-reported. Since the prevalence of self-reported tinnitus is very close to that of diagnosed tinnitus (severe tinnitus: 2.55% (2.45–2.65), *n* = 92,287; ICD_9–10_ H93.1: 2.77% (2.65–2.89), *n* = 74,351), diagnosed tinnitus may be used as a proxy of severe tinnitus. Hence, the use of national registry data would thus allow addressing the above-mentioned limitations with adjustments for multiple variables likely to contribute to tinnitus and determine the genuine sibling risk for tinnitus.

## 5. Conclusions

In conclusion, we reveal a familial aggregation for various forms of tinnitus and more so for constant and severe tinnitus in which a greater recurrence risk is seen in female probands when compared to males. Further longitudinal studies will be needed to improve the present analysis and possibly expand to national registry data (case-control or cohort studies with detailed information on familial history of tinnitus), which have been shown extremely powerful in the design of tinnitus studies.

## Figures and Tables

**Table 1:** Prevalence table of tinnitus subtypes according to sex. 95% confidence intervals (CI) are shown between parentheses. The sample sizes for unilateral and constant tinnitus are reduced due to an age-matching procedure. This procedure ensures that the average age of the population and that of the family members in STOP do not differ substantially (see Supplementary Materials 1 for details).

	Bilateral	Unilateral	Constant	Severe
Total sample size	67,615	59,507	26,696	92,287
Both genders	8.49%	6.68%	7.38%	2.55%
	(8.28–8.70)	(6.48–6.88)	(7.70–770)	(2.45–2.65)
Males	10.79%	7.24%	10.79%	3.26%
	(10.45–11.15)	(6.94–7.56)	(10.45–11.15)	(3.1–3.44)
Females	6.56%	6.20%	6.56%	1.99%
	(6.31–6.82)	(5.94–6.47)	(6.31–6.82)	(1.87–2.11)

**Table 2:** Recurrence risk ratio (λs) for participants with tinnitus and affected siblings. T = tinnitus. Estimates in bold are statistically significant at 0.05 level. * demarks the groups for which an artificially aged population estimate reported in Table 1 was used to calculate λs. This procedure is detailed in Supplementary Materials 1.

Respondents (*n*)	Number of Relatives	Number of Relatives with Tinnitus	λs (95% CI)	*p* Value
*Both genders*				
Bilateral T (1480)	1211	184	1.79 (1.55–2.04)	<0.0001
Unilateral T (413) *	324	43	1.99 (1.45–2.56)	0.0001
Constant T (1751) *	1408	238	2.29 (2.01–2.58)	<0.0001
Severe T (361)	297	55	7.27 (5.56–9.07)	<0.0001
*Male*				
Bilateral T (756)	612	99	1.50 (1.23–1.78)	0.0001
Unilateral T (168) *	126	13	1.42 (0.75–2.20)	0.1344
Constant T (923) *	735	119	1.58 (1.31–1.86)	<0.0001
Severe T (171)	140	23	5.03 (3.22–7.01)	<0.0001
*Female*				
Bilateral T (724)	599	85	2.16 (1.74–2.60)	<0.0001
Unilateral T (245) *	198	30	2.44 (1.66–3.29)	<0.0001
Constant T (828) *	673	119	3.32 (2.75–3.92)	<0.0001
Severe T (190)	157	32	10.25 (7.14–13.61)	<0.0001

## Supplementary Materials

The following are available online at https://www.mdpi.com/2077-0383/9/12/3812/s1. Supplementary Materials 1: Description of the methodology to obtain confidence intervals for recurrence risk ratios. Supplementary Materials 2: Results from the familial aggregation in STOP.
