# Supplementary material for: Sex-Dependent Aggregation of Tinnitus in Swedish Families
Source: J Clin Med. 2020 Nov 25;9(12):3812. doi: 10.3390/jcm9123812 (PMC7760080; doi:10.3390/jcm9123812)
Supplement: Supplementary file 1 [file jcm-09-03812-s001.zip › jcm-971608 SUPP conversion.pdf]

Supplementary Materials 1

Confidence Intervals for Recurrence Risk Ratios

1. Theoretical Background

The recurrence risk ratio λ derived as the ratio of prevalence of a condition in a group of family members (K_r) divided by the prevalence in the population K is a common measure for familial aggregation of a condition (disease). The measure λ and related generalizations have been reported in many scientific works in the past and recently [1,2]. The popularity of λ may likely be attributed to the simplicity of both its calculation and interpretation. Nevertheless, a potential drawback of λ is that the derivation of confidence intervals (CIs) is not straightforward. Therefore, this step is hence often omitted in practice, and the presented results consist only of point estimates [3–5]. The interpretability of these results is therefore limited, since the uncertainty of the point estimates is not taken into consideration.

From a statistical perspective, it is noteworthy that the term "population" should not be interpreted in the statistical sense here, since this would mean that we possess all relevant information on the entire world population. Instead, we are dealing with two samples: the first represents the sample of probands, and the second one represents the general population. Thus, the number of affected relatives of affected probands can be interpreted as a random variable X_r generated by repeated, independent sampling with probability p_r. The parameter p_r corresponds to the (unknown true) probability of a relative of an affected proband being affected by the condition. Thus, with n_r denoting the sample size (the total number of relatives of affected probands), the total number of affected relatives follows a binomial distribution with parameters p_r and n_r. That is,

$$X_r = n_r K_r \sim Binomial(p_r, n_r) \quad (1)$$

A similar argumentation logic applies to the quantity K. With p (as well unknown) and n corresponding to probability of being affected by the condition in the general population and the population size, respectively, holds

$$X = nK \sim Binomial(p, n) \quad (2)$$

when considering the number of people with the condition as a random variable X. Consequently, in order to derive the properties of λ, one may use that

$$\frac{n_r}{n}\lambda = \frac{n_r}{n}\frac{K_r}{K} = \frac{X_r}{X} \Leftrightarrow \lambda = \frac{X_r}{n_r} \Big/ \frac{X}{n}. \quad (3)$$

Re-writing λ this way permits to apply the results of Katz et al. (1978) [6]. Assuming independence of the random variables X_r and X, these authors showed that log (λ) approximately follows a normal distribution, i.e.

$$\log(\lambda) \sim N\left(\log\left(\frac{p_r}{p}\right), \frac{1-p_r}{x_r} + \frac{1-p}{x}\right) \quad (4)$$

Since these first results, other authors have followed and presented alternative ways for deriving confidence intervals for ratios of two binomial variables. To name only a few established results: a skewness correction was proposed [7,8], investigated confidence intervals based on Likelihood Scores [9] and employ the power divergence family for improved accuracy. Moreover, there is a rich literature on the properties CIs for recurrence risk in the context of logistic-type regression settings [10,11]. It should be noted, however, that these regression approaches usually assume the population prevalence to be constant, which allows to model the variability of the sample prevalence with complex modeling approaches.

While many advancements have certainly been made during the past years, the applicability of the very large majority of results relies on certain distributional assumptions. These are often only fulfilled for sufficiently large samples, where the required size for each method needs to be

investigated in simulation settings. An alternative approach is a re-sampling technique known as bootstrapping, which we describe in the following.

2. The Bootstrap

The bootstrap (or: bootstrapping) corresponds to a collection of computing-intensive methods which can be used to examine the properties of statistics such as λ in cases where their theoretical and asymptotical behavior is unclear or difficult to derive. The basic idea behind all bootstrap techniques is to determine the sampling distribution of the statistic by repetitive re-sampling. The literature related to bootstrapping is large and has grown substantially in the past decades. For a short overview of bootstrap fundamentals [12–14].

Re-sampling plays a core role for the bootstrap, and in the basic implementations one usually encounters either the "non-parametric" or the "parametric" bootstrapping. Both variations have the same goal: evaluating the properties a statistic by computing it for a high number of so-called "bootstrap samples". For the non-parametric case, the re-sampling is carried out by drawing observations with replacement from the original sample. Parametric sampling works by first fitting some kind of parametric model (distribution) to the original sample, and then sampling from this fitted model (distribution). In order to determine properties of our quantity λ, we need to carry out two sampling procedures, since the number of affected probands as well as the uncertainty in the population prevalence possess impact on the variability of λ. As will be shown later, non-parametric and parametric bootstrapping both leads to identical results for λ, which is usually not the case.

3. Bootstrap Confidence Intervals and p-values

A variety of approaches exists for deriving CIs via bootstrapping. Those most commonly used are basic, percentile, Studentized, bias-corrected, and accelerated bootstrap. We employ the percentile because of its appealing simplicity and ease of implementation, which permits us to set up this bootstrap variant straightforwardly in all common programming languages. However, one should keep in mind that more appropriate alternatives exist if the sampling distribution is highly skewed, or when sample sizes are very small. In order to carry out the percentile bootstrap, the following steps are carried out successively:

1. Generate B bootstrap samples by either parametric or non-parametric bootstrapping. In our case, each bootstrap sample consists of two sub-samples: one for re-sampling the number of affected probands X_r, and one for re-sampling the prevalence in the general population via X.
2. For each bootstrap sample, calculate corresponding estimate of λ (given by $\hat{\lambda} = \frac{x_r}{n_r} / \frac{x}{n}$). These estimates are denoted by $\hat{\lambda}^{*(b)}$, $b = 1, \ldots, B$.
3. Derive the CI for $\hat{\lambda}$ by $\left(\hat{\lambda}^{*}_{\alpha/2}, \hat{\lambda}^{*}_{1-\alpha/2}\right)$. Here, $\hat{\lambda}^{*}_{\alpha/2}$ and $\hat{\lambda}^{*}_{1-\alpha/2}$ denote the $\alpha/2$ and $1 - \alpha/2$ percentile, respectively, of the sample of bootstrapped values $\hat{\lambda}^{*(b)}$, $b = 1, \ldots, B$.

After carrying out the Steps 1. and 2. above, p-values can also directly calculated from the distribution of the $\hat{\lambda}^{*(b)}$. To test the null hypothesis that λ is lambda is smaller or equal to one, one simply needs to count the number of bootstrap estimates which fall into this interval. Table 2 reports p-values from a one-sided hypothesis.

4. Implementation of the Non-Parametric Bootstrap

In the following we present the most relevant aspects for implementing the non-parametric bootstrap described above. Section 6 below contains R code for a toy example with detailed comments. The first step consists of setting up the necessary parameters. We chose the severe tinnitus group with both genders:

```
# population
n <- 92287
x <- 2352
```

```
p <- x / n
# probands
n.r <- 297
x.r <- 55
p.r <- x.r / n.r
```

The quantities `n`, `x`, and `p` correspond to the population size, the number of cases, and prevalence, respectively. The quanties `n.r`, `x.r`, and `p.r` are defined analogously for the probands. In order to carry sample from the population and sample, we need to create these sets named `pop` and `samp` first.

```
pop <- rep(c(0, 1), c(n - x, x))
samp <- rep(c(0, 1), c(n.r - x.r, x.r))
```

Then, the loop carrying out the bootstrap loop looks as follows:

```
for (i in 1 : n.bs.samp) {
   prop.pop.bs[i] <- sum(sample(pop,
                      size = n,
                      replace = TRUE)) / n
   prop.samp.bs[i] <- sum(sample(samp,
                              size = n.r,
                              replace = TRUE)) / n.r
   lambda.bs[i] <- prop.samp.bs[i] / prop.pop.bs[i]
}
```

The vectors `prop.pop.bs`, `prop.samp.bs`, and `lambda.bs` collect the bootstrapped values for the proportion of cases (i.e. prevalence) in the population, the proportion of cases in the sample, and values of λ, respectively. The 95% CI for λ then results directly from the empirical quantiles calculated by

```
quantile(lambda.bs, c(0.025, 0.975))
```

It is noteworthy that this this way of implementing the bootstrap is computationally inefficient, since the sampling procedure is carried out in a rather primitive way. For illustrative purposes we set the number of bootstrap samples to 10^3, which is in general a too low number for a percentile bootstrap. If one increases the value, it quickly becomes clear that there is room for improvement.

5. Implementation of the Parametric Bootstrap

Instead of sampling from the sets `pop` and `samp`, the parametric bootstrap uses the integrated R function `rbinom` to carry out an equivalent sampling procedure. The bootstrap loop then looks as follows.

```
for (i in 1 : n.bs.samp) {
   prop.pop.bs[i] <- rbinom(n = 1,
                         size = n,
                         prob = p) / n
   prop.samp.bs[i] <- rbinom(n = 1,
                          size = n.r,
                          prob = p.r) / n.r
   lambda.bs[i] <- prop.samp.bs[i] / prop.pop.bs[i]
}
```

This approach has the advantage that it is computationally much more efficient then the non-parametric version. In our example script, we increase the number of bootstrap samples from 10^3 to 10^5, but need much less computational time for executing the loop. Nevertheless, this sampling can again be further improved by directly sampling `n = n.bs.samp` observations with the `rbinom` function, thus avoiding the loop altogether. That is, the above part is replaced by

```
prop.pop.bs <- rbinom(n = n.bs.samp,
                      size = n,
```

```
                              prob = p) / n
    prop.samp.bs <- rbinom(n = n.bs.samp,
                               size = n.r,
                               prob = p.r) / n.r
```

With this version, increasing the number of bootstrap samples from 10^5 to 10^7 results in a computational time between the non-parametric version and the first parametric bootstrap implementation. Since 10^7 bootstrap samples yield very stable and reproducible results, we chose this value for the calculation of all CIs.

6. Toy Example

The following lines demonstrate the implementation of a percentile bootstrap using the statistical software R. The code can either be copy-pasted to a separate .r-file or directly into the R console.

```
##################################################
# toy example for percentile bootstrap           #
# to obtain ci's of lambda                       #
##################################################

library(Hmisc)

# example used here: severe, both genders
# define / calculate all quantities required for the
different bootstraps
# population
n <- 92287
x <- 2352
p <- x / n # 2.55%
# probands
n.r <- 297
x.r <- 55
p.r <- x.r / n.r # ~18.5%
# check: point estimate of lambda, population confidence
interval
p.r / p
100 * binconf(x, n, alpha = 0.05, method = "wilson")

# initializing the random number generator for
reproducibility of results
RNGversion("3.6.0")
set.seed(533)



# non-parametric bootstrap
# ------------------------

# generate the samples to draw from
pop <- rep(c(0, 1), c(n - x, x))
samp <- rep(c(0, 1), c(n.r - x.r, x.r))
```

```
# number of bootstrap samples, proportions in the population
/ sample of probands, and lambdas
n.bs.samp <- 10 ^ 3 # increase if results are unstable
prop.pop.bs <- rep(NA, n.bs.samp)
prop.samp.bs <- rep(NA, n.bs.samp)
lambda.bs <- rep(NA, n.bs.samp)

# loop over number of bootstrap samples
for (i in 1 : n.bs.samp) {
   prop.pop.bs[i] <- sum(sample(pop,
                        size = n,
                        replace = TRUE)) / n
   prop.samp.bs[i] <- sum(sample(samp,
                                size = n.r,
                                replace = TRUE)) / n.r
   lambda.bs[i] <- prop.samp.bs[i] / prop.pop.bs[i]
}

# visual inspection of the results
par(mfrow = c(3, 1), mgp = c(2, 0.5, 0))
t.breaks <- 20
hist(prop.pop.bs, breaks = t.breaks, prob = TRUE, xlim =
c(0.02, 0.03))
hist(prop.samp.bs, breaks = t.breaks, prob = TRUE, xlim =
c(0, 0.4))
hist(lambda.bs, breaks = t.breaks, prob = TRUE, xlim = c(0,
12))
abline(v = quantile(lambda.bs, c(0.025, 0.05, 0.5, 0.975)),
col = "red")
abline(v = 1, lty = 2, col = "blue")
legend("topright",
       legend = c("lambda = 1", "Q0.025, Q0.05, Q0.5,
Q0.975"),
       col = c("blue", "red"),
       lty = c(2, 1))

# 2.5% and 97.5% quantiles for the 95% confidence interval,
as well as the median and 5% quantile
quantile(lambda.bs, c(0.025, 0.05, 0.5, 0.975))

# one-sided p-value
sum(lambda.bs <= 1) / n.bs.samp



# parametric bootstrap v1
# ----------------------

# number of bootstrap samples, proportions in the population
/ sample of probands, and lambdas
n.bs.samp <- 10 ^ 5 # increase if results are unstable
```

```
prop.pop.bs <- rep(NA, n.bs.samp)
prop.samp.bs <- rep(NA, n.bs.samp)
lambda.bs <- rep(NA, n.bs.samp)

# loop over number of bootstrap samples
for (i in 1 : n.bs.samp) {
   prop.pop.bs[i] <- rbinom(n = 1,
                            size = n,
                            prob = p) / n
   prop.samp.bs[i] <- rbinom(n = 1,
                             size = n.r,
                             prob = p.r) / n.r
   lambda.bs[i] <- prop.samp.bs[i] / prop.pop.bs[i]
}

# inspection of the results
mean(lambda.bs)
par(mfrow = c(3, 1), mgp = c(2, 0.5, 0))
t.breaks <- 20
hist(prop.pop.bs, breaks = t.breaks, prob = TRUE, xlim =
c(0.02, 0.03))
hist(prop.samp.bs, breaks = t.breaks, prob = TRUE, xlim =
c(0, 0.4))
hist(lambda.bs, breaks = t.breaks, prob = TRUE, xlim = c(0,
12))
quantile(lambda.bs, c(0.025, 0.05, 0.5, 0.975))
abline(v = quantile(lambda.bs, c(0.025, 0.05, 0.5, 0.975)),
col = "red")
abline(v = 1, lty = 2, col = "blue")
legend("topright",
       legend = c("lambda = 1", "Q0.025, Q0.05, Q0.5,
Q0.975"),
       col = c("blue", "red"),
       lty = c(2, 1))



# parametric bootstrap v2
# -----------------------

# number of bootstrap samples, proportions in the population
/ sample of probands, and lambdas
n.bs.samp <- 10 ^ 7 # increase if results are unstable
prop.pop.bs <- rep(NA, n.bs.samp)
prop.samp.bs <- rep(NA, n.bs.samp)
lambda.bs <- rep(NA, n.bs.samp)

# vectorized operation instead of loop
prop.pop.bs <- rbinom(n = n.bs.samp,
                      size = n,
                      prob = p) / n
```

```
prop.samp.bs <- rbinom(n = n.bs.samp,
                       size = n.r,
                       prob = p.r) / n.r
lambda.bs <- prop.samp.bs / prop.pop.bs

# inspection of the results
mean(lambda.bs)
par(mfrow = c(3, 1), mgp = c(2, 0.5, 0))
t.breaks <- 30
hist(prop.pop.bs, breaks = t.breaks, prob = TRUE, xlim =
c(0.02, 0.03))
hist(prop.samp.bs, breaks = t.breaks, prob = TRUE, xlim =
c(0, 0.4))
hist(lambda.bs, breaks = t.breaks, prob = TRUE, xlim = c(0,
12))
quantile(lambda.bs, c(0.025, 0.05, 0.5, 0.975))
abline(v = quantile(lambda.bs, c(0.025, 0.05, 0.5, 0.975)),
col = "red")
abline(v = 1, lty = 2, col = "blue")
legend("topright",
       legend = c("lambda = 1", "Q0.025, Q0.05, Q0.5,
Q0.975"),
       col = c("blue", "red"),
       lty = c(2, 1))
```

7. Robustness of Recurrence Risk Ratio Estimates Towards Population Age and Age of Family Members

The estimation of λ implicitly requires that family members should be representative for the population considered. In the best case, the family members are the result of a (stratified) random sampling procedure. This is, , however, not the case for our data. Since tinnitus is suject to a higher prevalence among older persons, it is of particular importance that the age distribution of the family members and the population, respectively, are relatively similar or in the best case identical. Otherwise, the estimate of λ could be biased: for example, one woud most likely over-estimate λ if the family members were consistently older than the population.

In our setting, the age of the family members (i.e. siblings) is unknown. Therefore, we assume that the siblings are of the same age as the probands. This leads to the average age shown in the first two columns of Table 1.

Table 1. Average age of the participants, all individuals belonging to the full population, and members of the artificially aged population. T = tinnitus.

	Average age (years)		
**Respondents**	**Siblings**	**Full population**	**Aged population**
Both genders			
Bilateral T (1480)	49.659	49.861	
Unilateral T (413)	53.336	49.861	53.293
Constant T (1751)	51.672	42.466	54.546
Severe T (361)	49.659	53.331	
Male			
Bilateral T (756)	49.678	49.934	
Unilateral T (168)	51.732	49.934	53.195
Constant T (923)	50.908	43.362	54.719
Severe T (171)	49.678	53.932	
Female			
Bilateral T (724)	49.640	49.800	
Unilateral T (245)	54.380	49.800	53.376
Constant T (828)	52.514	41.875	54.424
Severe T (190)	49.640	52.859	

It is visible that the average age of the siblings and of the general population roughly correspond to each other for bilateral, and severe tinnitus. Nevertheless, the members of our familial sample are about nine years younger than the population for constant tinnitus. This results from the comparably high proportion of young subjects in the LifeGene cohort. In addition, siblings with unilateral tinnitus are almost four years younger than the population. Hence, relying our analysis on the full population would very likely lead to an overestimation of λ.

In order to avoid such a bias, we chose to "artificially age" our population for the cases of constant and unilateral tinnitus. To achieve this, we exclude all individuals younger than 40 from the population with constant tinnitus, and individuals younger than 30 from the population with unilateral tinnitus. This comparably pragmatic approach is necessary because age information of parts of our population is only available in 10-year blocks. Furthermore, the inclusion of age as covariate is not possible in our model, since we cannot apply the above-mentioned logistic-type regression approaches.

The last column of Table 1 displays the average age of our modified population, which serves for deriving the estimates of λ. For unilateral tinnitus, sample and population age roughly correspond to each other. The siblings for the constant tinnitus sample are a bit younger than the population, resulting in a rather conservative λ estimate. It may be noted that the population size reduces from 51832 to 26696 in the constant tinnitus case (male: 20594 to 11,051; female: 31238 to 15,645). The impact of this reduction on the estimate of λ is limited, because all these numbers are still comparably large. For unilateral tinnitus, the reduction is even less important (all: 67615 to 59507; male: 30748 to 27233; female: 36867 to 32274).

Table 2 shows the estimation results for the original as well as the aged population for constant and unilateral tinnitus. As anticipated, all lambda estimates decrease, which underlines the necessity of basing the calculus on an appropriate population. However, all conclusions remain the same.

Table 2. Recurrence risk ratio (λs) for participants with unilateral or constant tinnitus bases on the full and aged, respectively, population. T = tinnitus. Estimates in bold are statistically significant at 0.05 level.

	Full population		Aged population	
**Respondents**	**λs (95% CI)**	***p* value**	**λs (95% CI)**	***p* value**
Both genders				
Unilateral T (413)	2.14 (1.56 - 2.75)	**<0.0001**	1.99 (1.45 – 2.56)	**0.0001**
Constant T (1751)	3.61 (3.18 – 4.06)	**<0.0001**	2.29 (2.01 – 2.58)	**<0.0001**
Male				
Unilateral T (168)	1.52 (0.81 – 2.36)	0.0972	1.42 (0.75 – 2.20)	0.1344
Constant T (923)	2.46 (2.04 – 2.89)	**<0.0001**	1.58 (1.31 – 1.86)	**<0.0001**
Female				
Unilateral T (245)	2.64 (1.80 – 3.55)	**<0.0001**	2.44 (1.66 – 3.29)	**<0.0001**
Constant T (828)	5.16 (4.29 – 6.08)	**<0.0001**	3.32 (2.75 – 3.92)	**<0.0001**

References

1. Chen WJ, Liu PH, Ho YY, Chien KL, Lo MT, Shih WL; et al. Sibling recurrence risk ratio analysis of the metabolic syndrome and its components over time. BMC genetics. 2003;4 Suppl 1:S33. Epub 2004/02/21, doi:10.1186/1471-2156-4-S1-S33. PubMed PMID: 14975101; PubMed Central PMCID: PMCPMC1866469.
2. Rybicki BA, Elston RC. The relationship between the sibling recurrence-risk ratio and genotype relative risk. American journal of human genetics. 2000;66(2):593-604. Epub 2000/03/21, doi:10.1086/302778. PubMed PMID: 10677319; PubMed Central PMCID: PMCPMC1288112.
3. Requena T, Espinosa-Sanchez JM, Cabrera S, Trinidad G, Soto-Varela A, Santos-Perez S; et al. Familial clustering and genetic heterogeneity in Meniere's disease. Clinical genetics. 2014;85(3):245-52. Epub 2013/03/26, doi:10.1111/cge.12150. PubMed PMID: 23521103.
4. Alarcon-Segovia D, Alarcon-Riquelme ME, Cardiel MH, Caeiro F, Massardo L, Villa AR; et al. Familial aggregation of systemic lupus erythematosus, rheumatoid arthritis, and other autoimmune diseases in 1,177 lupus patients from the GLADEL cohort. Arthritis and rheumatism. 2005;52(4):1138-47. Epub 2005/04/09, doi:10.1002/art.20999. PubMed PMID: 15818688.
5. Esposito F, Guaschino C, Sorosina M, Clarelli F, Ferre L, Mascia E; et al. Impact of MS genetic loci on familial aggregation, clinical phenotype, and disease prediction. Neurol Neuroimmunol Neuroinflamm. 2015;2(4):e129. Epub 2015/07/18, doi:10.1212/NXI.0000000000000129. PubMed PMID: 26185776; PubMed Central PMCID: PMCPMC4503410.
6. Katz D, Baptista J, Azen SP, Pike MC. Obtaining Confidence Intervals for the Risk Ratio in Cohort Studies. Biometrics. 1978;34(3):469-74.
7. Gart JJ, Nam J. Approximate Interval Estimation of the Ratio of Binomial Parameters: A Review and Corrections for Skewness. Biometrics. 1988;44(2):323-38.
8. Nam J. Confidence Limits for the Ratio of Two Binomial Proportions Based on Likelihood Scores: Non-Iterative Method. Biometrical Journal. 1995;37(3):375-9.
9. Bedrick EJ. A Family of Confidence Intervals for the Ratio of Two Binomial Proportions. Biometrics. 1987;43(4):993-8.
10. Mitry D, Williams L, Charteris DG, Fleck BW, Wright AF, Campbell H. Population-based estimate of the sibling recurrence risk ratio for rhegmatogenous retinal detachment. Investigative ophthalmology & visual science. 2011;52(5):2551-5. Epub 2011/01/20, doi:10.1167/iovs.10-6375. PubMed PMID: 21245406.
11. Fotouhi A, Etemadi A, Hashemi H, Zeraati H, Bailey-Wilson JE, Mohammad K. Familial aggregation of myopia in the Tehran eye study: Estimation of the sibling and parent offspring recurrence risk ratios. Br J Ophthalmol. 2007;91(11):1440-4. Epub 2007/05/15, doi:10.1136/bjo.2007.120162. PubMed PMID: 17494955; PubMed Central PMCID: PMCPMC2095425.
12. Efron B. The Bootstrap and Modern Statistics. Journal of the American Statistical Association. 2000;95(452):1293-6.
13. Efron B, Tibshirani RJ. An Introduction to the Bootstrap. Hall/CRC Ca, editor1994.
14. Hall P. The Bootstrap and Edgeworth Expansion. Statistics SSi, editor1992.

.
